# Development and validation of a machine learning-based predictive model for secondary post-tonsillectomy hemorrhage

**DOI:** 10.3389/fsurg.2023.1114922

**Published:** 2023-02-07

**Authors:** Xiandou Hu, Zixuan Yang, Yuhu Ma, Mengqi Wang, Weijie Liu, Gaoya Qu, Cuiping Zhong

**Affiliations:** ^1^ The First School of Clinical Medicine, Gansu University of Chinese Medicine, Lanzhou, China; ^2^ Otolaryngology Head and Neck Surgery, The 940th Hospital of Joint Logistics Support Force of Chinese People's Liberation Army, Lanzhou, China; ^3^ The First School of Clinical Medicine, Lanzhou University, Lanzhou, China; ^4^ School of Clinical Medicine, Ningxia Medical University, Yinchuan, China

**Keywords:** chronic tonsillitis, tonsillectomy, secondary hemorrhage, machine learning, predictive model

## Abstract

**Background:**

The main obstacle to a patient's recovery following a tonsillectomy is complications, and bleeding is the most frequent culprit. Predicting post-tonsillectomy hemorrhage (PTH) allows for accurate identification of high-risk populations and the implementation of protective measures. Our study aimed to investigate how well machine learning models predict the risk of PTH.

**Methods:**

Data were obtained from 520 patients who underwent a tonsillectomy at The 940th Hospital of Joint Logistics Support Force of Chinese People's Liberation Army. The age range of the patients was 2–57 years, and 364 (70%) were male. The prediction models were developed using five machine learning models: decision tree, support vector machine (SVM), extreme gradient boosting (XGBoost), random forest, and logistic regression. The performance of the models was evaluated using the area under the receiver operating characteristic curve (AUC). Shapley additive explanation (SHAP) was used to interpret the results of the best-performing model.

**Results:**

The frequency of PTH was 11.54% among the 520 patients, with 10.71% in the training group and 13.46% in the validation set. Age, BMI, season, smoking, blood type, INR, combined secretory otitis media, combined adenoidectomy, surgical wound, and use of glucocorticoids were selected by mutual information (MI) method. The XGBoost model had best AUC (0.812) and Brier score (0.152). Decision curve analysis (DCA) showed that the model had a high clinical utility. The SHAP method revealed the top 10 variables of MI according to the importance ranking, and the average of the age was recognized as the most important predictor variable.

**Conclusion:**

This study built a PTH risk prediction model using machine learning. The XGBoost model is a tool with potential to facilitate population management strategies for PTH.

## Introduction

Tonsillectomy is one of the most common surgical procedures in otolaryngology-head and neck. Over 600,000 tonsil-related treatments are reportedly carried out annually across the globe (1). However, postoperative complications remain the greatest challenge for patient recovery, occurring in about 5%–15% of cases, the most common of which is related to post-tonsillectomy hemorrhage (PTH) (1, 2). The incidence of PTH is approximately 1%–19%. PTH is regarded as a primary cause of death related with tonsillectomy and has been suggested to be a potentially lethal complication, particularly in children (3, 4). Anemia and hemodynamic abnormalities, which can hinder the patient’s recovery and even necessitate repeat surgery, are possible PTH hazards that could have a negative psychological impact on the patient as well as physical harm. Sarny et al. found that the reoperation rate after tonsillectomy was 4.6 percent in a multicenter cohort of 9,405 adult and pediatric patients in Austria, with PTH being the most significant complication (5, 6). Clinical doctors should conduct studies and be concerned with determining how to find the risk factors and reduce PTH.

Previous investigations have recorded the characteristics and the prevalence of PTH (6). Age, gender, surgical indication, surgical method, perioperative drugs (including NSAIDs and glucocorticoids), and seasonal fluctuations are only a few of the many factors associated with PTH (7–10). The causes of PTH risk, however, are currently very highly contentious. Therefore, accurate risk prediction for PTH and excellent identification of high-risk individuals will give clinicians the required direction for prevention and treatment (11).

In recent years, artificial intelligence has been widely used to explore early warning predictions for many disease complications. Machine learning models and advanced statistical tools can be used to predict the outcome of complex datasets based on iterative learning, thereby making these models more accurate and stable through the selection of variable features. Therefore, more researchers advocate the use of new predictive models based on machine learning to support personalized treatment for patients (11–13). To the best of our knowledge, there is no predictive model for PTH based on machine learning.

This study used machine learning method to develop and validate prediction models, to provide individualized management for the postoperative prognosis of patients.

## Materials and methods

Patient studies were conducted according to the guiding principles of the Helsinki Declaration and were approved by the Ethics Committee of the 940th Hospital of Joint Logistics Support Force of Chinese People’s Liberation Army (2022KYLL165).

### Study population

**Figure 1:**
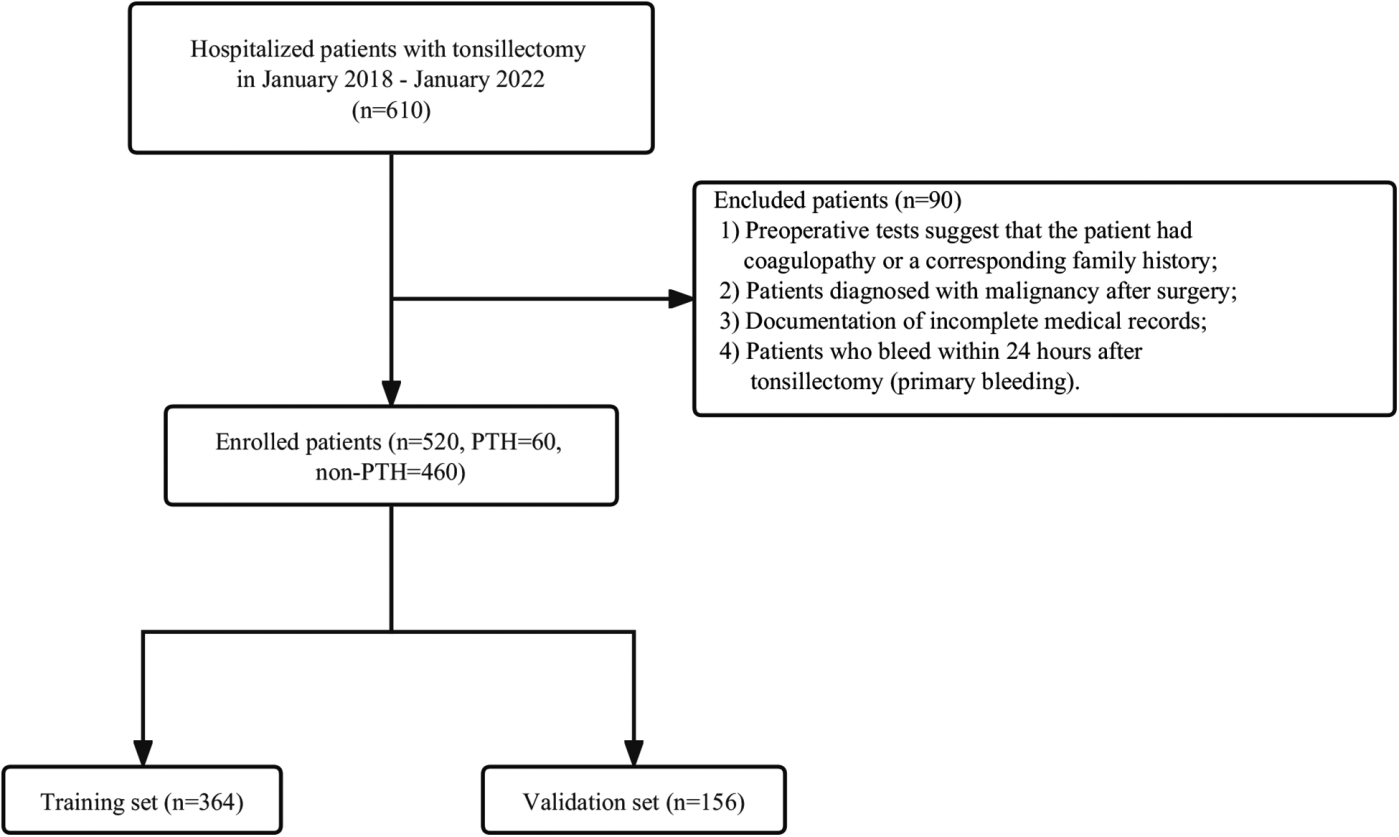
Flowchart of patient selection. Non-PTH, No Post-tonsillectomy hemorrhage; PTH, Post-tonsillectomy hemorrhage.

This study collected patients who visited our otorhinolaryngology-head and neck surgery department for tonsillectomy from January 2018 to January 2022. The exclusion criteria were as follows: (1) Preoperative tests suggest that the patient had coagulopathy or a corresponding family history; (2) Patients diagnosed with malignancy after surgery; (3) Documentation of incomplete medical records;4. Patients who bleed within 24 h after tonsillectomy (primary bleeding). Figure 1 presents the inclusion and exclusion criteria as well as the patient-select flowchart. In total 520 patients were identified, including 60 PTH cases and 460 non-PTH cases. A random number generated by the computer was allocated to the training group (*n* = 364) and validation group (*n* = 156) in a ratio of 7:3.

### Study variables

Risk prediction models were created using 27 variables selected from medical reports and published literature. Including (1) Basic patient information: sex, age, BMI, smoking, alcohol drinking, nation, address, season, tonsillitis attack per year, combined secretory otitis media, combined allergic rhinitis, diagnosis, tonsil grading, pharyngeal congestion, use of glucocorticoids, use of atomization inhalation, combined adenoidectomy, surgical wound, whether tonsillar pillars were sutured, pre-operative infection, and post-operative infection. (2) laboratory test: blood type, fibrinogen, activated partial thromboplastin time (APTT), prothrombin time (PT), thrombin time (TT), international normalized ratio (INR). Multiple imputations were used for missing data. The data was assumed to be missing at random, fully conditional imputation was performed using R software version 4.0.4. Smote oversampling of the data was performed using python version 3.9.0.

### Model development and evaluation

The variable selection used the mutual information (MI) method, and created a binary outcome model for the prediction of the risk of PTH based on the selected predictors, a binary outcome for the prediction model was defined as the presence or absence of PTH. To develop predictive models used five machine learning methods (decision trees, support vector machines (SVM), extreme gradient boosting (XGBoost), random forests, and logistic regression). The receiver operating characteristic (ROC) curve, area under the curve (AUC), accuracy, sensitivity, specificity, positive predictive value (PPV), and negative predictive value (NPV) were used to evaluate the predictive power of the model. In addition, decision curve analysis (DCA) was performed to assess the utility of the decision model by quantifying the net benefit under different threshold probabilities. The interpretation of the prediction model was performed by SHAP, a unified approach that precisely calculates the contribution and impact of each variable on the final prediction (12). Each observation in the dataset can be interpreted by a specific SHAP value.

### Statistical analysis

Statistical analysis using Python version 3.9.0 and R software version 4.0.4. Continuous variables were expressed as the median and interquartile range (IQR) or mean and standard deviation (SD), and categorical variables were expressed as frequencies and percentages. Mann–Whitney *U* test and Independent Samples *t*-test were performed to compare continuous variables. Fisher's exact test and *χ*^2^ test were used to compare categorical variables. A two-sided *P-*value <0.05 indicated that the difference was statistically significant.

## Results

### Patient characteristics

520 patients were included in this analysis. Among them, the age distribution of the patients was 2–57 years, 364 (70%) patients were male. The incidence of PTH was 11.54%, 10.71% in the training set, and 13.46% in the validation set. The clinical characteristics of the training and validation sets were not statistically significantly different. Baseline information and clinical characteristics of the patients were shown in Table 1.

**Table 1:** Clinical characteristics in the training and validation sets.

	Training set			Validation set			*P (Inter)*
	Non-PTH (*N* = 325)	PTH (*N* = 39)	*P(Intra)*	Non-PTH (*N* = 135)	PTH (*N* = 21)	*P(Intra)*	
Sex			0.092			1	0.491
Male	219 (67.4%)	32 (82.1%)		98 (72.6%)	15 (71.4%)		
Female	106 (32.6%)	7 (17.9%)		37 (27.4%)	6 (28.6%)		
Nation			0.043			0.429	0.975
Han nationality	296 (91.1%)	31 (79.5%)		123 (91.1%)	18 (85.7%)		
Other nationalities	29 (8.92%)	8 (20.5%)		12 (8.89%)	3 (14.3%)		
Address			0.438			0.780	0.319
Urban area	176 (54.2%)	18 (46.2%)		66 (48.9%)	9 (42.9%)		
Rural district	149 (45.8%)	21 (53.8%)		69 (51.1%)	12 (57.1%)		
Season			0.054			0.143	0.143
Spring	72 (22.2%)	6 (15.4%)		17 (12.6%)	5 (23.8%)		
Summer	121 (37.2%)	15 (38.5%)		61 (45.2%))	11 (52.4%)		
Autumn	23 (7.08%)	8 (20.5%)		9 (6.67%))	2 (9.52%)		
Winter	109 (33.5%)	10 (25.6%)		48 (35.6%))	3 (14.3%)		
Smoking			<0.001			0.083	0.096
No	305 (93.8%)	27 (69.2%)		119 (88.1%)	15 (71.4%)		
Yes	20 (6.15%)	12 (30.8%)		16 (11.9%)	6 (28.6%)		
Alcohol drinking			0.079			0.207	0.189
No	313 (96.3%)	35 (89.7%)		126 (93.3%)	18 (85.7%)		
Yes	12 (3.69%)	4 (10.3%)		9 (6.67%)	3 (14.3%)		
Tonsillitis attack per year			0.249			0.196	0.498
None	117 (36.0%)	10 (25.6%)		52 (38.5%)	5 (23.8%)		
1–4	157 (48.3%)	40 (51.3%)		58 (43.0%)	9 (42.9%)		
5–8	28 (8.62%)	3 (7.69%)		16 (11.9%)	3 (14.3%)		
≥9	23 (7.08%)	6 (15.4%)		9 (6.67%)	4 (19.0%)		
Secretory otitis media			0.022			0.695	1
No	290 (89.2%)	39 (100%)		121 (89.6%)	20 (95.2%)		
Yes	35 (10.8%)	0 (0.00%)		14 (10.4%)	1 (4.76%)		
Allergic rhinitis			0.320			0.201	0.935
No	282 (86.8%)	31 (79.5%)		117 (86.7%)	16 (76.2%)		
Yes	43 (13.2%)	8 (20.5%)		18 (13.3%)	5 (23.8%)		
Diagnosis			0.048			0.083	0.844
Chronic tonsillitis	97 (29.8%)	19 (48.7%)		36 (26.7%)	10 (47.6%)		
Obstructive sleep apnea	9 (2.77%)	1 (2.56%)		4 (2.96%)	1 (4.76%)		
All	219 (67.4%)	19 (48.7%)		95 (70.4%)	10 (47.6%)		
Blood Type			0.225			0.004	0.231
A	104 (32.0%)	8 (20.5%)		37 (27.4%)	1 (4.76%)		
B	95 (29.2%)	10 (25.6%)		53 (39.3%)	5 (23.8%)		
O	91 (28.0%)	17 (43.6%)		33 (24.4%)	13 (61.9%)		
AB	35 (10.8%)	4 (10.3%)		12 (8.89%)	2 (9.52%)		
Pharyngeal congestion			0.064			0.806	0.274
No	164 (50.5%)	13 (33.3%)		59 (43.7%)	8 (38.1%)		
Yes	161 (49.5%)	26 (66.7%)		76 (56.3%)	13 (61.9%)		
Tonsil size			0.102			0.008	0.003
I	15 (4.62%)	2 (5.13%)		11 (8.15%)	7 (33.3%)		
II	173 (53.2%)	27 (69.2%)		59 (43.7%)	7 (33.3%)		
III	137 (42.2%)	10 (25.6%)		65 (48.1%)	7 (33.3%)		
Use of glucocorticoids			0.356			0.250	0.369
No	57 (17.5%)	4 (10.3%)		30 (22.2%)	2 (9.52%)		
Yes	268 (82.5%)	35 (89.7%)		105 (77.8%)	19 (90.5%)		
Use atomization inhalation			0.808			0.381	0.646
No	153 (47.1%)	17 (43.6%)		69 (51.1%)	8 (38.1%)		
Yes	172 (52.9%)	22 (56.4%)		66 (48.9%)	13 (61.9%)		
Adenoidectomy			0.001			0.055	0.984
No	123 (37.8%)	26 (66.7%)		50 (37.0%)	13 (61.9%)		
Yes	202 (62.2%)	13 (33.3%)		85 (63.0%)	8 (38.1%)		
Surgical wound			0.002			0.004	0.530
Satisfactory	240 (73.8%)	19 (48.7%)		98 (72.6%)	8 (38.1%)		
Unsatisfactory	85 (26.2%)	20 (51.3%)		37 (27.4%)	13 (61.9%)		
Add suture			0.396			0.293	0.891
Non-suture	312 (96.0%)	36 (92.3%)		129 (95.6%)	19 (90.5%)		
Suture	13 (4.00%)	3 (7.69%)		6 (4.44%)	2 (9.52%)		
Pre-operative infection			0.134			0.644	0.538
No	310 (95.4%)	35 (89.7%)		126 (93.3%)	19 (90.5%)		
Yes	15 (4.62%)	4 (10.3%)		9 (6.67%)	2 (9.52%)		
Post-operative infection			0.001			0.048	1
No	321 (98.8%)	34 (87.2%)		134 (99.3%)	19 (90.5%)		
Yes	4 (1.23%)	5 (12.8%)		1 (0.74%)	2 (9.52%)		
BMI	17.6[14.8;22.4]	20.8 [16.3;25.4]	0.017	17.4 [14.9;22.4]	20.2 [18.4;24.9]	0.014	0.535
Age	7.00 [5.00;18.0]	23.0[8.00;28.5]	<0.001	8.00 [5.00;20.5]	18.0[8.00;27.0]	0.01	0.962
FIB	2.32 (0.52)	2.32 (0.58)	0.981	2.36 (0.64)	2.25 (0.40)	0.27	0.795
APTT	27.9 (2.79)	29.0 (3.44)	0.055	27.6 (3.78)	28.2 (2.22)	0.295	0.499
PT	11.2 (0.66)	11.1 (0.58)	0.364	11.3 (1.90)	10.9 (0.58)	0.066	0.512
TT	18.5 (1.04)	18.4 (1.11)	0.598	18.9 (3.25)	18.4 (0.91)	0.148	0.091
INR	0.95 (0.08)	0.94 (0.05)	0.648	0.99 (0.24)	0.92 (0.05)	0.007	0.510

BMI, body mass index; FIB, fibrinogen; APTT, activated partial thromboplastin time; PT, prothrombin time; TT, thromboplastin time; INR, International Normalized Ratio. Unsatisfactory surgical wound: The white membrane of the tonsillar fossa was yellowed and thickened after the operation as observed by main surgery as documented in the medical record.

*P(Intra)* the result of uni-variable analyses between PTH and Non-PTH groups, *P(inter)* significant difference between training and validation set.

### Model building and evaluation

**Figure 2:**
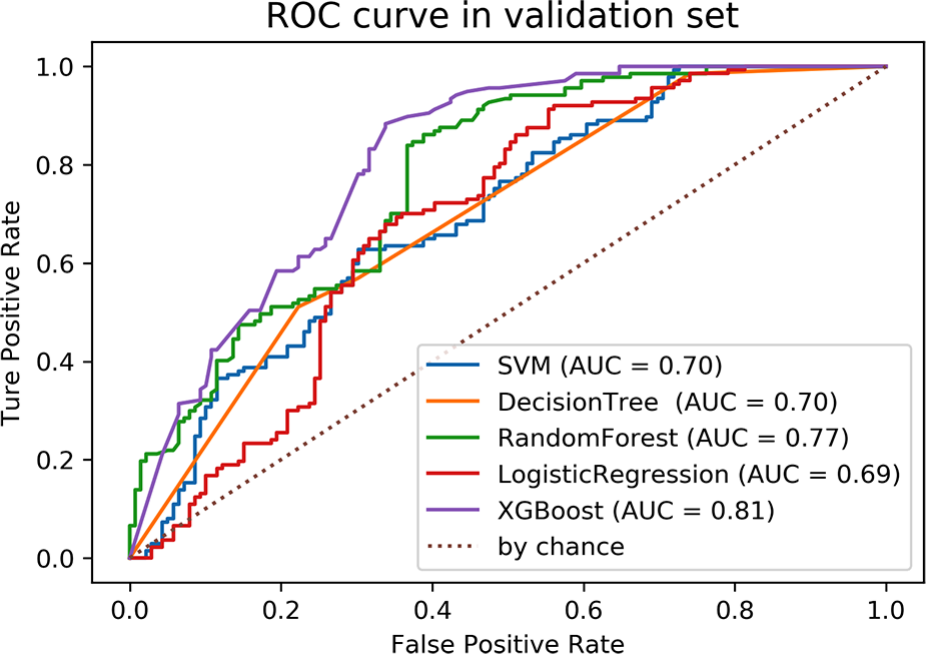
Evaluation of the five machine learning models based on the AUC of the ROC curve in validation set. AUC, area under the curve; ROC, receiver operating characteristic.

Variable selection was performed using the mutual information (MI) method, and 10 variables were screened, including Age, BMI, season, smoking, blood type, INR, combined secretory otitis media, combined adenoidectomy, surgical wound, and use of glucocorticoids. The 10 variables selected were entered using five machine learning models to predict the risk of PTH. The performance evaluation of the five machine learning models in validation set is shown in Table 2. The ROC curve of five machine learning models in the training set were shown in the Supplementary Figure S1. Compared to other models, the XGBoost model had the highest AUC (0.812), Specificity (0.788), positive predictive value (0.768) and negative predictive value (0.715), and its four hyperparameters were n_estimators = 200, min_samples_split = 20, min_samples_leaf = 10, max_depth = 2. While logistic regression has the worst generalization ability (AUC = 0.69) in validation set, as shown in Figure 2.

**Table 2:** Performance of predictive models generated by five machine learning models.

Model	AUC	Accuracy	Sensitivity	Specificity	NPV	PPV	F1 score
SVM	0.699	0.641	0.712	0.569	0.661	0.627	0.639
DT	0.698	0.644	0.777	0.511	0.693	0.617	0.638
RF	0.773	0.673	0.647	0.701	0.662	0.687	0.674
LR	0.689	0.670	0.640	0.701	0.658	0.685	0.670
XGBoost	0.812	0.739	0.690	0.788	0.715	0.768	0.783

PPV, positive predictive value; NPV, negative predictive value; SVM, support vector machines; DT, decision tree; RF, random forest; LR, logistic regression.

**Figure 3:**
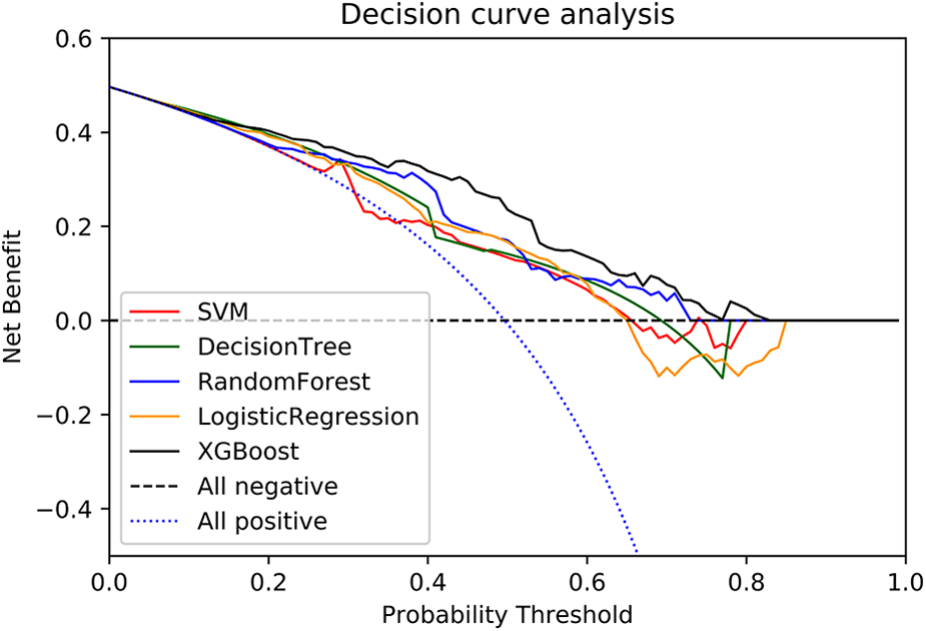
A decision curve was used to evaluate the clinical utility of models. The y axis shows net income. the x axis shows the threshold probability. Within a reasonable threshold probability range, the XGBoost model in the validation set achieved higher benefits.

DCA was performed on the five machine learning models in the validation set to compare the net benefit of the best model and clinical decision alternatives (Figure 3). Clinical net benefit is defined as the minimum probability of disease, when further intervention would be warranted. The plot measures the net benefit at different threshold probabilities (12). The blue line in Figure 3 indicates the hypothesis that all patients received the intervention, while the black line indicates that no patients received the intervention due to the heterogeneity of the study population. In contrast, the net benefits of the XGBoost model exceeded that of other models in the validation set.

**Figure 4:**
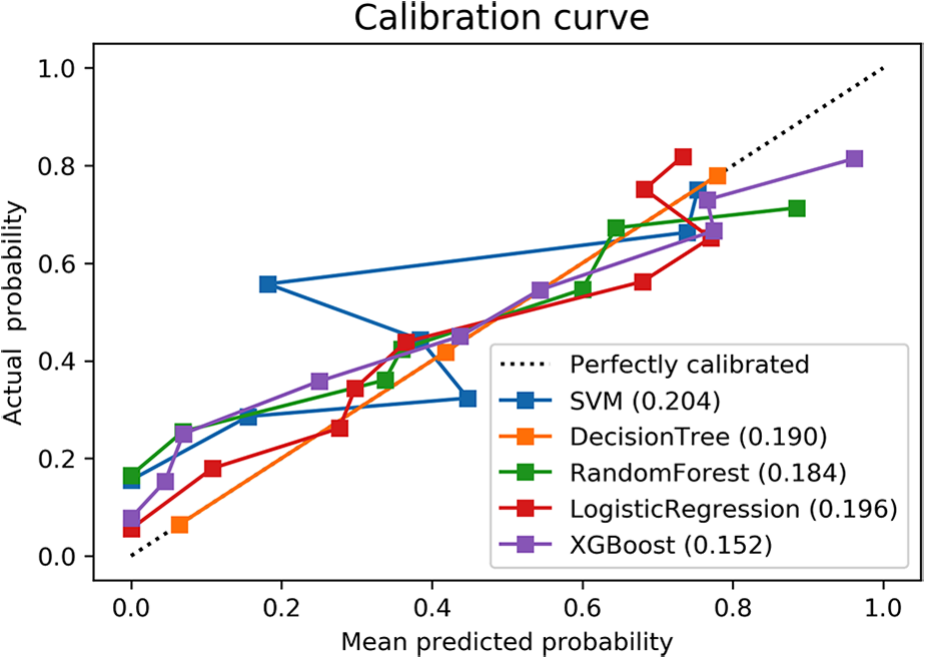
Calibration curves of five machine learning models in the validation set.

In addition, this study used the calibration curve Brier metric to evaluate the model performance (14–16). AUC measures the overall ability of the model to distinguish between high and low risk patients; however, it hardly reflects the performance of the model over the entire range of possible predictions. This defect is compensated for by its calibration curve, which is plotted to show the relation between the model prediction and the label (17). As in Figure 4, x-axis represents the probability of PTH calculated by the clinic model, and y-axis represents the actual rate of PTH. The diagonal dashed line indicates a perfect estimation of the ideal model, which means that the estimated result is the same as the actual result. The solid line indicates the performance of the clinical model, and its close alignment with the diagonal dashed line indicates a good estimate. It can be seen that the XGBoost model is better than the others, with a Brier score of 0.152.

### Explanation of risk factors

**Figure 5:**
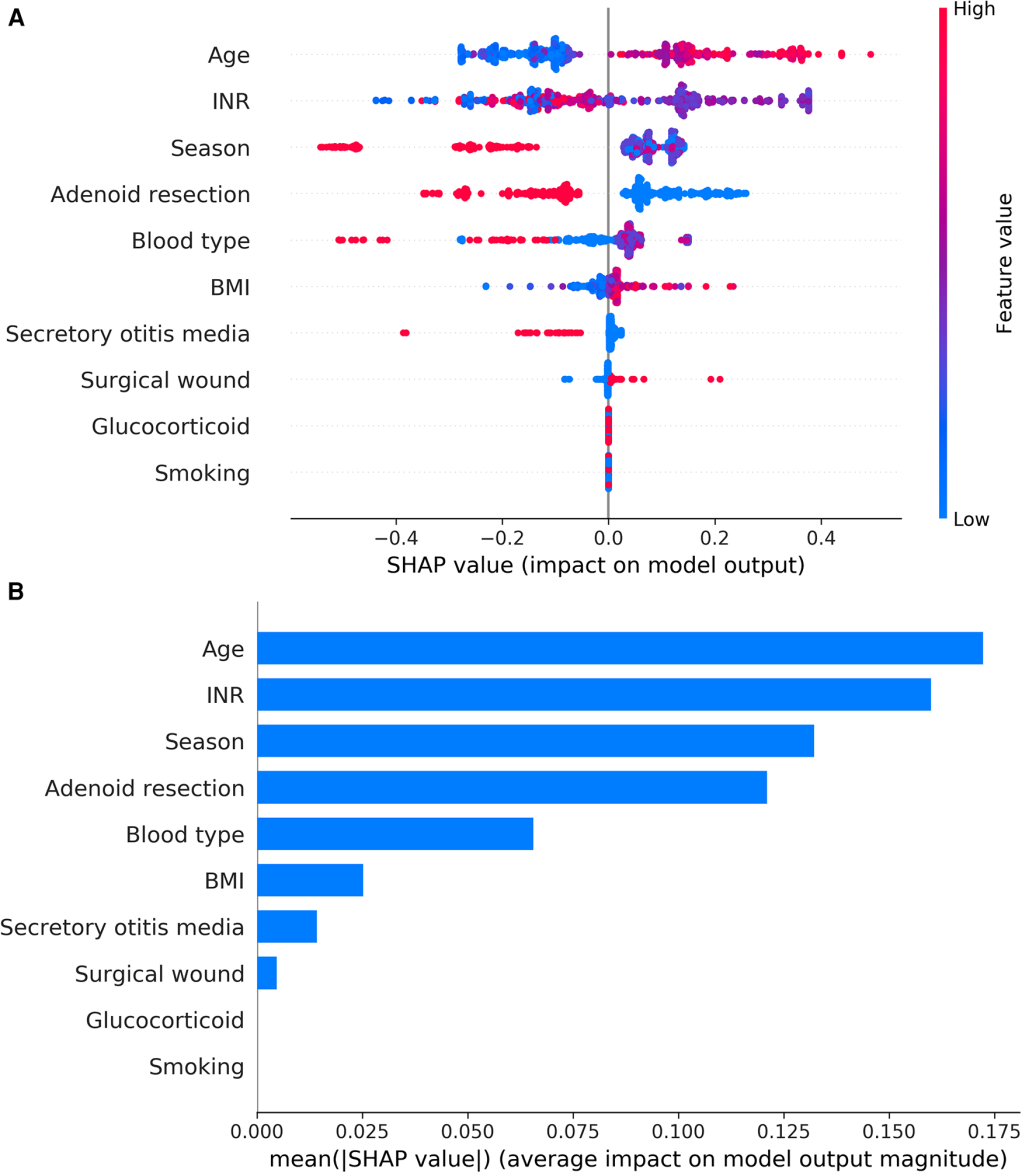
(**A**) SHAP summary plot of clinical features. Each feature of each patient was colored by a point according to an attribute value. Red represents a higher value, and blue represents a lower value. (**B**) shows the importance matrix plot of the XGBoost model, describing the importance of each variable in predicting PTH. Adenoid resection: adenoidectomy.

The results of the XGBoost model were interpreted with SHAP by calculating the contribution of each variable to the prediction. The SHAP summary plot and the importance matrix plot of the XGBoost model were shown in Figure 5. The SHAP summary plot (Figure 5A) had one point for each feature per patient based on the estimated value, where red represents the higher value and blue represents the lower value. The horizontal coordinate is the SHAP value. The larger shape indicated that the feature had a high predictive value for the risk of PTH in a given sample. Importance matrix plots depicted the importance of each variable in predicting the risk of PTH (Figure 5B). In summary, the top 10 characteristics in descending order of importance were: age, INR, season, combined with adenoidectomy, blood type, BMI, combined with secretory otitis media, surgical wound, use of glucocorticoids, and smoking.

### Applying predictive models

**Figure 6:**
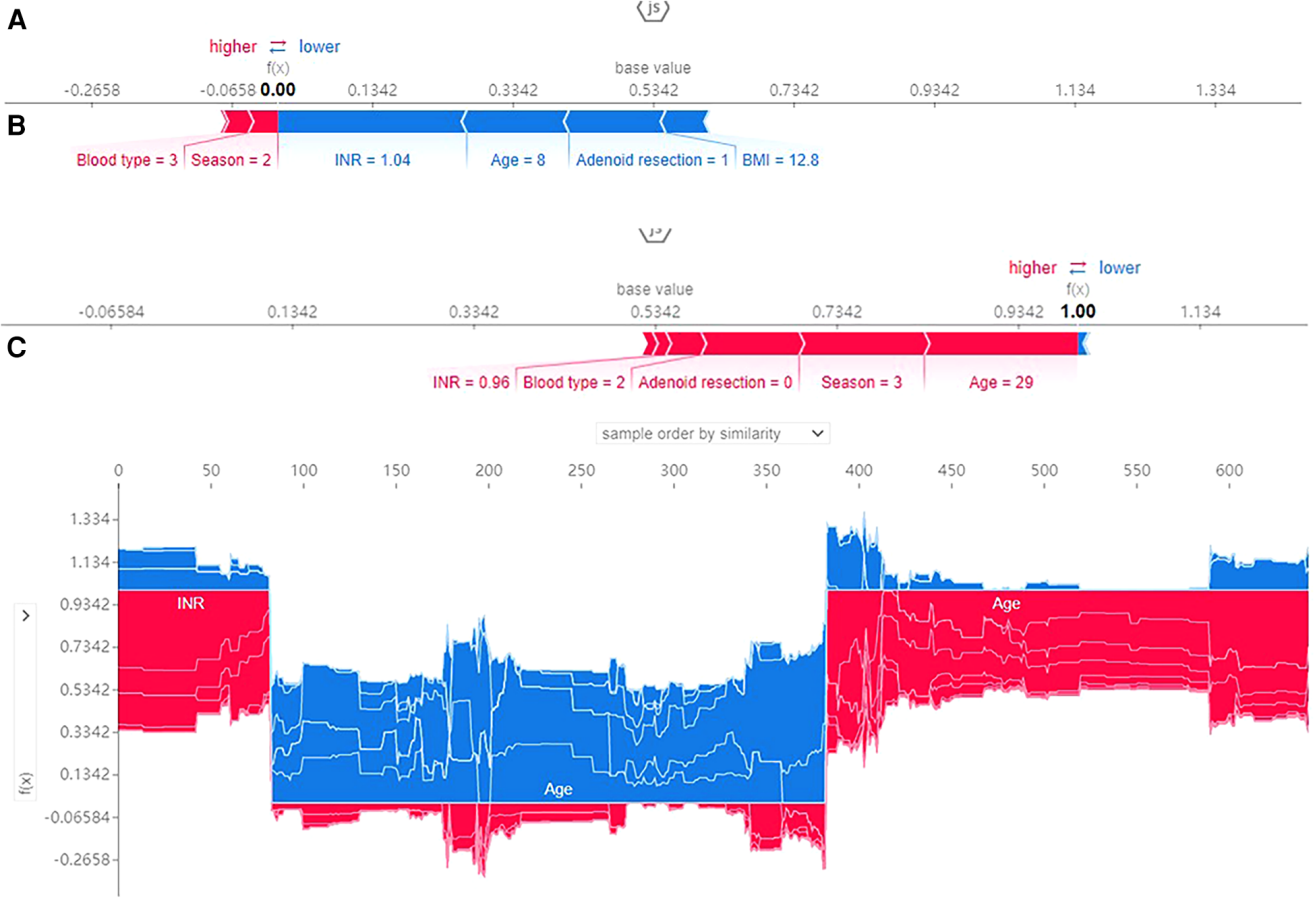
SHAP force plot for patients in the dataset at high (**A**) or low (**B**) risk of PTH; 1, 2, 3 and 4 represents spring, summer, autumn, winter and blood type A, B, O, AB; (**C**) SHAP values (global interpretation) for the training set. The abscissa represents each patient, and the ordinate represents the SHAP value.

SHAP force plots illustrated profiles of patients at high or low risk for developing an outcome and showed how a predictive model can facilitate personalized treatment planning. SHAP force plots for the XGBoost model were shown in Figure 6. SHAP values represent the relevant predictive features of an individual patient and the contribution of each feature to PTH prediction. Red indicates features with PTH risk and blue indicates features without PTH risk. The length of the arrow helps to realize the size of the predicted effect. The longer the arrow, the larger the effect. Figure 6A showed an 8-year-old patient with a predicted PTH probability of 24.6%. Figure 6B showed a 29-year-old patient with a predicted PTH probability of 91.3%. Figure 6C showed the parameters with negative influence in blue and those with positive influence in red. The advantage of this graph was that it gave a clear combination of parameters that contribute greatly to the model.

## Discussion

According to previous studies, the risk factors associated with PTH were more controversial. At this stage, despite the understanding of PTH and the implementation of the concept of post-tonsillectomy management in many centers, there are still some unknowns and regional differences that cause hemorrhage. Due to the increasing need for cost-effectiveness in healthcare systems, patterns of postoperative care and clinical decisions making also vary greatly in different countries and institutions. To the authors' knowledge, there has been little development of PTH prediction models. Accurate prediction of prognosis is crucial for patient-centered care, whether to guide inclusive decision-making or select treatment strategies. The application of machine learning to medical and clinical conditions forms a major emerging research trend (18). In this study, predictive risk factors for PTH were identified using the mutual information method and risk prediction models for PTH were created. The performance of five machine learning models was compared. The results showed that the XGBoost model had the highest AUC, positive predictive value, and negative predictive value. The SHAP method further explained the predictors and model prediction performance. It also provided a simple and robust method for personalized prediction of PTH, which can provide important information for medical decision support.

For an overview of the XGBoost logic, this non-linear machine learning model used the input (independent) variables in the training dataset to construct an array of decision trees in every possible combination to establish a series of thresholds that split variables to maximize the information gain (19). In our study, the XGBoost model predicted patient risk with excellent calibration and good validation. Previous reports have described machine learning techniques as “black boxes” that provide little information on how predictions are made. This reduces clinician acceptance because they are reluctant to make medical diagnoses based on opaque decisions (20). We used the SHAP method to explain the decision-making process of the XGBoost model. In clinical practice, visualization of SHAP can be translated into clinical insight. It can show the accuracy and complexity of the prediction model better and provide some reference for postoperative patient care and personalized decision-making treatment (14).

Among the several important factors explained by SHAP, age has been confirmed to be a risk factor for PTH. Compared with children, adult patients have a higher risk of PTH. Some studies suggest that the postoperative bleeding rate of adults is as high as 8.6%–14.5%, while that of children is about 2.1%-5% (4, 20). A reasonable explanation is that adults with long-lasting chronic tonsillitis are prone to fibrosis of the tonsillar bed and that dissection of the tonsils can make surgery more difficult (21). The results of a previous retrospective study involving 897 patients showed that INR ≥ 1.2 as a risk factor helped to identify patients at higher risk of hemorrhage (4). INR was also identified as an important predictor in our study. This is consistent with previous studies. In addition, many past studies have shown a correlation between season and increased risk of PTH (22). It has been suggested that higher altitude and lower atmospheric moisture enhance the incidence of mucosal surface hemorrhage (23). In this study, the bleeding rate of patients increased in the spring. Our hospital is located in a high-altitude area in northwest China, where spring is relatively dry. It is regrettable that more detailed atmospheric data information was not further obtained in this study. However, it is necessary for the season to be used as an important factor to predict the PTH. In addition, some studies suggested that obesity, smoking, blood type, and perioperative hormone use were all important factors affecting the incidence of PTH (24–27). It had even been suggested that patients undergoing tonsillectomy must quit smoking to avoid bleeding after tonsillectomy. It is worth mentioning that in this study, we believed that the wound of the tonsillar fossa after surgery played a role. Our study found an increased rate of postoperative hemorrhage in patients with thicker and yellowing tonsillar fossa wounds after tonsillectomy. Few studies have investigated trauma as a potential factor. Therefore, we believe that attention should be paid to patients with yellowing of the tonsillar fossa and thickened white membranes after tonsillectomy.

This study has several strengths. Firstly, our study created and validated the first PTH risk prediction model by machine learning methods. Second, we included 10 risk factors, including surgical wound, and season, which were easy to be ignored. Third, our model can be used by clinicians and nurses as an intuitive way to predict PTH risk for the appropriate management of high-risk patients. Finally, this model can provide a new idea for future PTH research.

Our study has some limitations. The sample size in this study was small. Although we performed internal validation, external validation is needed to obtain more clinical evidence. The machine learning model in this study used the presence of combined secretory otitis media and adenoidectomy as predictor variables, but the number of adults with these diseases was small, so there was some confounding bias, and further studies are still needed to develop prediction models for different age groups. In addition, some scholars believe that primary bleeding is usually attributed to the type of surgical technique, and primary and secondary bleeding needs to be carefully distinguished and studied separately (28). The coblation technique is used for all tonsillectomies in our hospital. Therefore, the study focuses on the secondary hemorrhage. Furthermore, although the SHAP model can determine the influence of patient characteristics on prediction, this is only a mapping relationship between predictor variables and prediction outcomes, rather than a causal relationship (29). Despite these limitations, we developed the XGBoost model as a tool that has the potential to facilitate individualized treatment strategies for patients.

## Conclusion

In conclusion, we identified baseline demographic and clinical variables as predictive risk factors for PTH and used machine learning to construct a PTH risk prediction model for patients after tonsillectomy. The XGBoost model could efficiently improve the predictive accuracy of PTH. With increasing use in medicine, machine learning models could be considered for inclusion in clinical pathway decisions for clinical use and further validation. Future research will explore additional machine learning models applicable to predicting PTH risk.

## Data Availability

The original contributions presented in the study are included in the article/**Supplementary Material**, further inquiries can be directed to the corresponding author/s.
