# Supplementary figures and images for: Development and validation of a machine learning-based predictive model for secondary post-tonsillectomy hemorrhage
Source: Front Surg. 2023 Feb 7;10:1114922. doi: 10.3389/fsurg.2023.1114922 (PMC9941337; doi:10.3389/fsurg.2023.1114922)

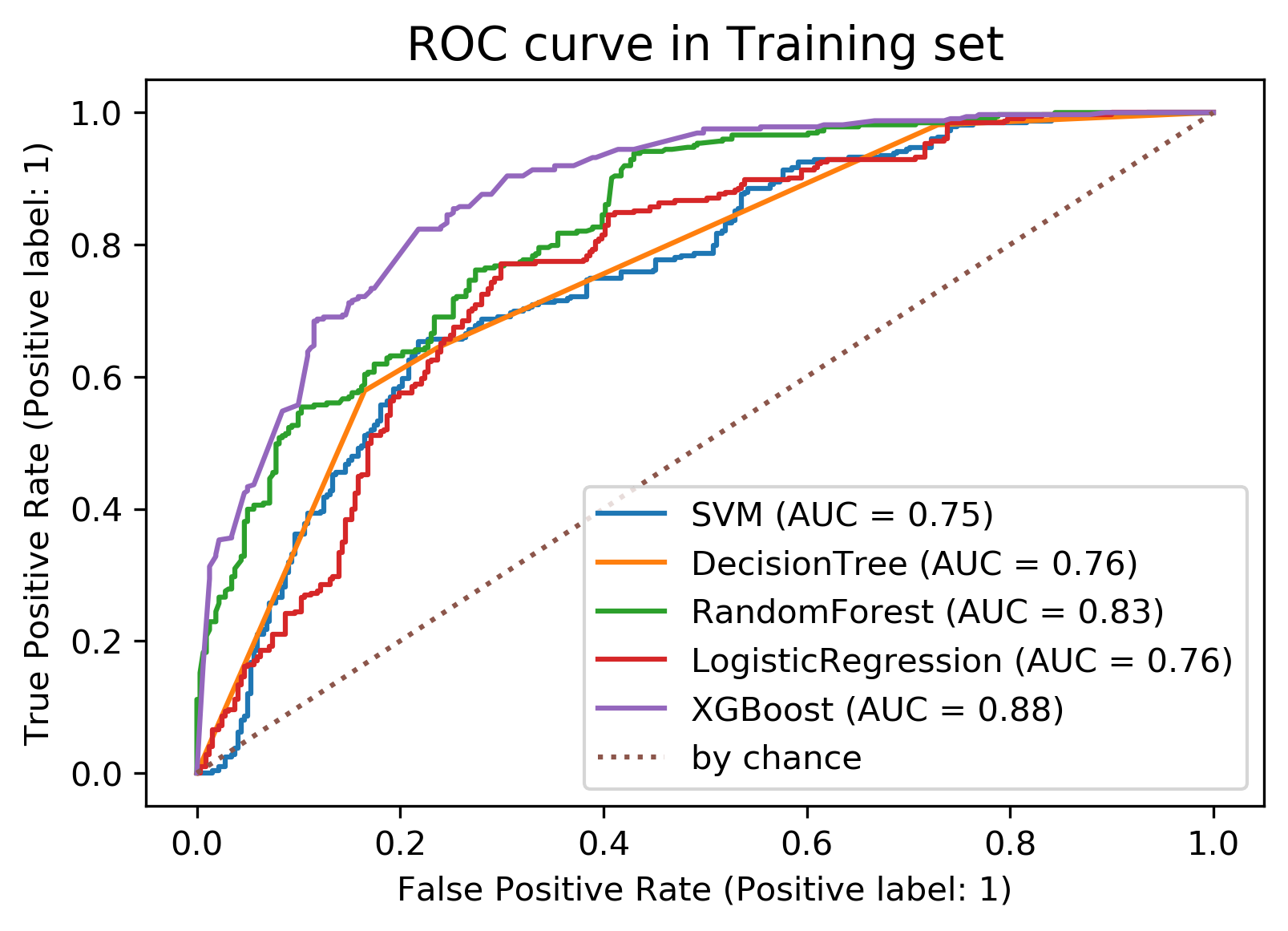

Supplement: Supplementary file 1 [file Image1.png]
